# Specific and redundant activities of *ETV1* and *ETV4* in prostate cancer aggressiveness revealed by co-overexpression cellular contexts

**DOI:** 10.18632/oncotarget.2847

**Published:** 2015-02-14

**Authors:** Diana Mesquita, João D. Barros-Silva, Joana Santos, Rolf I. Skotheim, Ragnhild A. Lothe, Paula Paulo, Manuel R. Teixeira

**Affiliations:** ^1^ Department of Genetics and Cancer Genetics Group – CI-IPOP, Portuguese Oncology Institute, Porto, Portugal; ^2^ Department of Cancer Prevention, Institute for Cancer Research, The Norwegian Radium Hospital, Oslo University Hospital, Nydalen, Oslo, Norway; ^3^ Centre for Cancer Biomedicine, Faculty of Medicine, University of Oslo, Oslo, Norway; ^4^ Department of Pathology and Molecular Immunology, Institute of Biomedical Sciences Abel Salazar (ICBAS), University of Porto, Portugal

**Keywords:** prostate cancer, *ETV1* and *ETV4* co-overexpression, target genes, oncogenic role, PEA3-positive tumors

## Abstract

Genomic rearrangements involving ETS transcription factors are found in 50–70% of prostate carcinomas. While the large majority of the rearrangements involve *ERG*, around 10% involve members of the PEA3 subfamily (*ETV1*, *ETV4* and *ETV5*). Using a panel of prostate cancer cell lines we found co-overexpression of ETV1 and ETV4 in two cell line models of advanced prostate cancer (MDA-PCa-2b and PC3) and questioned whether these PEA3 family members would cooperate in the acquisition of oncogenic properties or show functional redundancy. Using shRNAs we found that ETV1 and ETV4 have partially overlapping functions, with ETV1 being more relevant for cell invasion and ETV4 for anchorage-independent growth. *In vitro* expression signatures revealed the regulation of both specific and shared candidate targets that may resemble cellular mechanisms *in vivo* by interaction with the same intermediate partners. By combining the phenotypic impact data and the gene expression profiles of *in vitro* models with clinico-pathological features and gene expression profiles of ETS-subtyped tumors, we identified a set of eight genes associated with advanced stage and a set of three genes associated with higher Gleason score, supporting an oncogenic role of ETV1 and ETV4 overexpression and revealing gene sets that may be useful as prognostic markers.

## INTRODUCTION

Chromosomal rearrangements involving different members of the ETS family of transcription factors have been found to occur in prostate carcinomas (PCa) [1, 2]. The most prevalent gene fusion is the *TMPRSS2-ERG*, present in nearly half of all human prostate cancers, followed by rearrangements involving the *ETV1*, *ETV4*, *ETV5* and *FLI1* genes, often with promoter fusion partners other than *TMPRSS2* [3–10]. These rearrangements, ultimately leading to overexpression of 5′ truncated or full-length ETS transcription factors, are considered to be early molecular events in prostate carcinogenesis, not least because they are also found in around 20% of the pre-cancerous lesion high-grade prostate intraepithelial neoplasia [11]. However, *in vivo* and *in vitro* data have shown controversial results regarding the oncogenic role of *ERG* overexpression, with some studies suggesting that this ETS protein can induce the initiation of neoplastic transformation through the development of pre-invasive lesions [12, 13], whereas others reinforce the need of additional genomic alterations to drive cancer progression [14, 15].

Being part of a large family of 28 members of transcription factors, all sharing the characteristic DNA binding motif GGAA/T [16], it has been questioned whether ETS proteins have redundant or specific functions. Despite the fact that mutual exclusivity of ETS rearrangements is the rule (at least at the cellular level) in prostate carcinomas [6, 17] and the impact on cell invasion shared by *ERG* and *ETV1* [12, 18, 19], ETS members show both lack of tissue specificity and co-expression within a tissue [20–22]. Interestingly, our group has recently shown that the ETS transcription factors *ERG* and *ETV1* are able to regulate both specific and shared sets of genes in prostate cancer cells [23].

The low frequency of rearrangements involving *ETV4*, *ETV5* and *FLI1* (less than 2%), and the lack of cell line models harboring rearrangements of these ETS members, resulted in scarce knowledge of their oncogenic roles in prostate carcinogenesis. *In vitro* studies have implicated *ETV5* in cell invasion [3, 24–26], alike to *ERG* and *ETV1*, while *ETV4* seems to be required for both proliferation and anchorage-independent growth [27, 28]. On the other hand, co-expression of PEA3 family members (*ETV1*, *ETV4* and *ETV5*) is described to occur in several organs, both during embryonic development and in adult tissues [21, 22]. In 2012, two studies from the same group showed that both ERG and PEA3 rearrangements are found in metastatic lesions, but while ERG rearrangements seem to follow the positiveness seen in the primary tumor, PEA3 rearrangements can occur as clonal events during progression [29, 30]. Moreover, *in vivo* studies have shown that overexpression of these ETS members can occur during and promote prostate cancer progression [31, 32]. Considering these observations and the high homology between *ETV1* and *ETV4* (defined by their DNA binding motif), we questioned whether these PEA3 family members would cooperate or have redundant roles in prostate carcinogenesis, by regulating the same or distinct target genes and pathways, and whether *in vitro* models of ETV1 and ETV4 overexpression could reveal markers of tumor aggressiveness. We used two prostate cancer cell lines (MDA-PCa-2b and PC3) harboring co-overexpression of *ETV1* and *ETV4* to gain insight into their biological role *in vitro*, and found both specific and shared candidate target genes that may play a role in tumor aggressiveness *in vivo*.

## RESULTS

### PC3 and MDA-PCa-2b prostate cancer cell lines as *in vitro* models to study the oncogenic role of ETS co-expression

**Figure 1:**
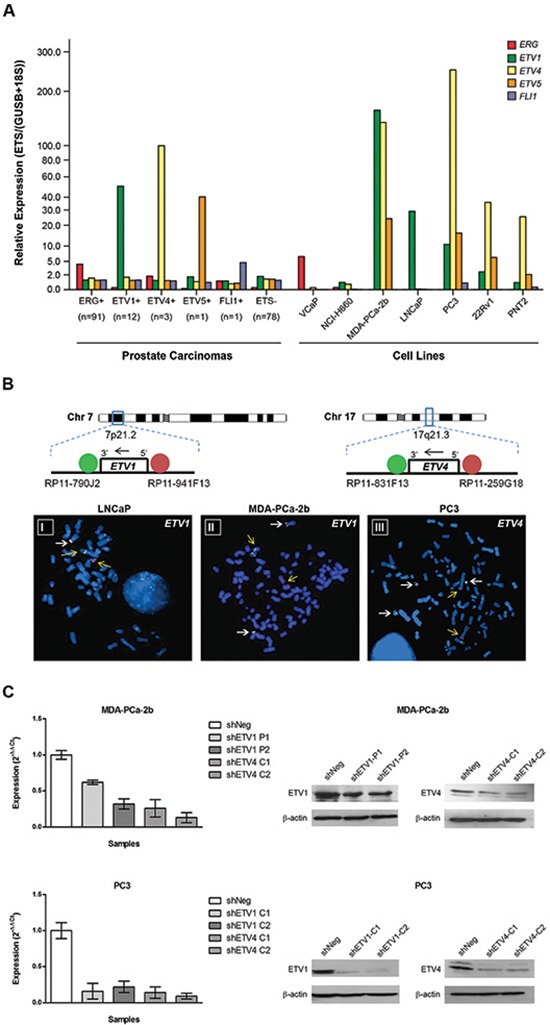
Characterization and establishment of the cell line models **(A)** Comparative expression levels of the five ETS transcription factors described to be involved in genomic rearrangements in PCa (*ERG*, *ETV1*, *ETV4*, *ETV5* and *FLI1*) in a panel of prostate carcinomas subtyped for ETS rearrangements and in prostate cell lines by TLDAs. Median-centered values of each ETS were obtained for each sample. For PCa subtypes the average values are shown. **(B)** FISH analysis for the *ETV1* locus in LNCaP and MDA-PCa-2b cell lines (I and II, respectively) and for the *ETV4* locus in PC3 cells (III). White arrows indicate normal co-localized signals and yellow arrows indicate gene rearrangements. **(C)** qRT-PCR and immunoblotting for *ETV1* and *ETV4* after stable silencing of each ETS in the MDA-PCa-2b and PC3 cell lines (*upper* and *lower* panels, respectively). A negative control (shNeg) and two independently silenced populations (*clonal*, C1 and C2, or *polyclonal*, P1 and P2) for the same target region were analyzed.

Using the TaqMan Low Density Array (TLDA) technology, we evaluated the expression of the five ETS genes known to be involved in genomic rearrangements in PCa (*ERG*, *ETV1*, *ETV4*, *ETV5* and *FLI1*) in a panel of cell lines representing various subtypes of prostate cancer (Figure 1A). Comparing with ETS rearrangement positive PCa, isolated overexpression of *ERG* was found in VCaP cells and isolated overexpression of *ETV1* was found in LNCaP cells, as reported by others [26]. Interestingly, the prostate carcinoma cell lines PC3 and MDA-PCa-2b exhibited a remarkable co-expression of the three members of the PEA3 subfamily of ETS transcription factors (*ETV1*, *ETV4* and *ETV5*). FISH analysis was performed in an attempt to find an explanation for the *ETV1* and *ETV4* outlier expressions in MDA-PCa-2b and PC3 cells. For that purpose, dual color break-apart probes flanking the 5′ and 3′ regions of each ETS were used. Although no *ETV1* rearrangement was found in the PC3 cell line (data not shown), MDA-PCa-2b and LNCaP cells showed chromosomal rearrangements involving the *ETV1* locus (7p21.2) (Figure 1B), as described by others [26]. Regarding *ETV4*, although no chromosomal rearrangement typical of a fusion gene involving this ETS was found, we were able to identify an atypical genomic rearrangement in the PC3 cell line which consisted in the presence of the 5′ region of the *ETV4* gene located in two distinct marker chromosomes, in addition to three chromosomes 17 with no rearrangements (Figure 1B). No rearrangement was found to mediate the aberrant overexpression of *ETV4* in MDA-PCa-2b cells (not shown). Considering the co-overexpression of *ETV1* and *ETV4* in MDA-PCa-2b and PC3 cells, we sought to dissect the role of these ETS in prostate carcinogenesis. Two previously established *ETV1* models of prostate carcinogenesis [23], namely with silencing and *de novo* expression of *ETV1* (in malignant LNCaP cells and benign PNT2 cells, respectively), were used as complementary cell line models uniquely overexpressing *ETV1*. We thus used MDA-PCa-2b and PC3 cells to establish two independent ETS downregulated cell populations for each cell line, which stably express shRNAs directed against one target region of *ETV1* or *ETV4* (explained in the Methods section), along with one negative control (shNeg). Quantitative RT-PCR and immunoblotting analyses showed an efficient decrease of either *ETV1* or *ETV4* in both MDA-PCa-2b cells (of around 40–60% and 70–80%, respectively) and PC3 cells (of around 70–80% and 80–90%, respectively) (Figure 1C), comparing with the shNeg controls. The *ETV1* expression levels of the previously established cell line models with significant silencing and *de novo* expression of *ETV1* (LNCaP and PNT2 cells, respectively) was previously shown [23]. The shETV1-LNCaP cells show a decrease of about 90–100% in *ETV1* expression while the PNT2-ETV1 cells used in this study show an increase in *ETV1* expression of about 90% of the expression present in LNCaP cells [23].

### Silencing of either ETV1 or ETV4 does not impact proliferation or apoptosis in a co-expression background

**Figure 2:**
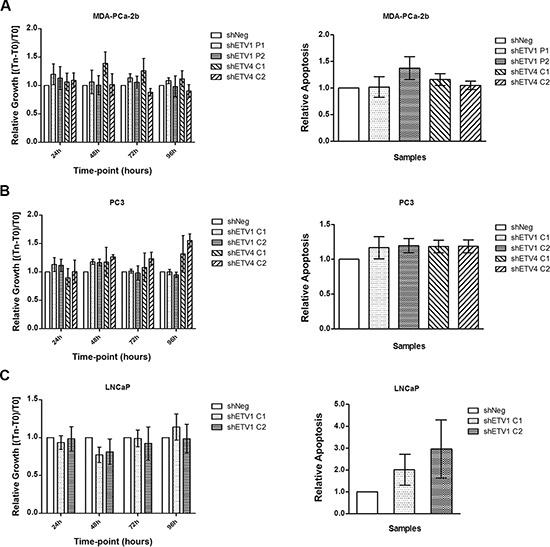
*In vitro* evaluation of the impact of ETV1 or ETV4 silencing in proliferation and apoptosis **(A–C)** Relative growth was estimated for four time-points in culture (*left*) and apoptosis for one time-point (96 hr, *right*), for the three cell line models with manipulated *ETV1* or *ETV4* expression: MDA-PCa2b **(A)**, PC3 **(B)** and LNCaP **(C)** cell lines. Results are shown for each silenced cell population relative to the shNeg cells from three independent experiments. There are no statistically significant results (*p* > 0.05).

To evaluate the possible effect of sustained *ETV1* or *ETV4* knockdown on PCa cell growth and apoptosis *in vitro* we used the MTT and the APOPercentage^TM^ assays, respectively. Silencing of *ETV1* or *ETV4* did not alter the growth rate or apoptosis levels in both ETS co-expressing models (MDA-PCa-2b and PC3) (Figure 2). Although a slight increase in apoptosis was observed, results do not reach statistical significance. In LNCaP cells, which only express *ETV1*, there is a clear tendency for higher apoptotic levels in the *ETV1* silenced populations, although statistical analysis show lack of significance (*p* > 0.05).

### ETV4 overcomes ETV1 in the regulation of anchorage-independent growth in a co-expression cellular context

**Figure 3:**
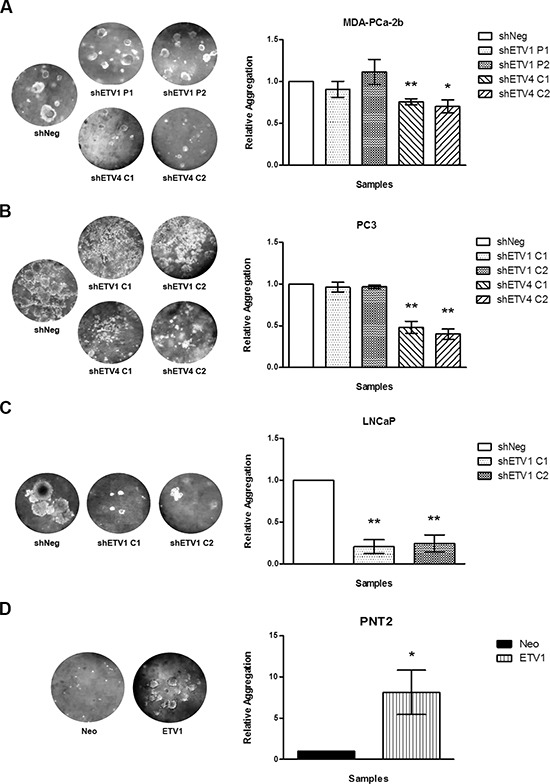
*In vitro* evaluation of the impact of ETV1 or ETV4 silencing in anchorage-independent growth **(A–D)** Qualitative visualization (*left*) and quantitative analysis (*right*) of the several cell line models with manipulated *ETV1* or *ETV4* expression: MDA-PCa-2b **(A)**, PC3 **(B)**, LNCaP **(C)**, and PNT2 **(D)** cell lines. Results are shown for each silenced cell population relative to the shNeg cells from three independent experiments. Statistically significant *p* values are showed by an asterisk (*p < 0.05; **p < 0.01).

To determine the involvement of *ETV1* and *ETV4* in the ability of cells to grow in the absence of an anchorage surface, associated with the metastasis process, we used an *in vitro* soft-agar assay. In contrast to shNeg cells, shETV4 cells showed a significant decrease in colony formation after two weeks in culture, originating 20% fewer colonies in MDA-PCa-2b cells and around 50–60% in PC3 cells (Figures 3A and 3B). No differences were observed in *ETV1* silenced cells from both cell line models. Contrarily, stable silencing of *ETV1* in LNCaP cells resulted in a highly significant decrease of more than 70% in the number of colonies formed (Figure 3C). An inverse effect was observed in PNT2 cells with *de novo ETV1* overexpression, showing nearly ten-fold more colonies than the respective control (Figure 3D).

### Knockdown of ETV1 inhibits cell invasion *in vitro* with a greater impact than knockdown of ETV4

**Figure 4:**
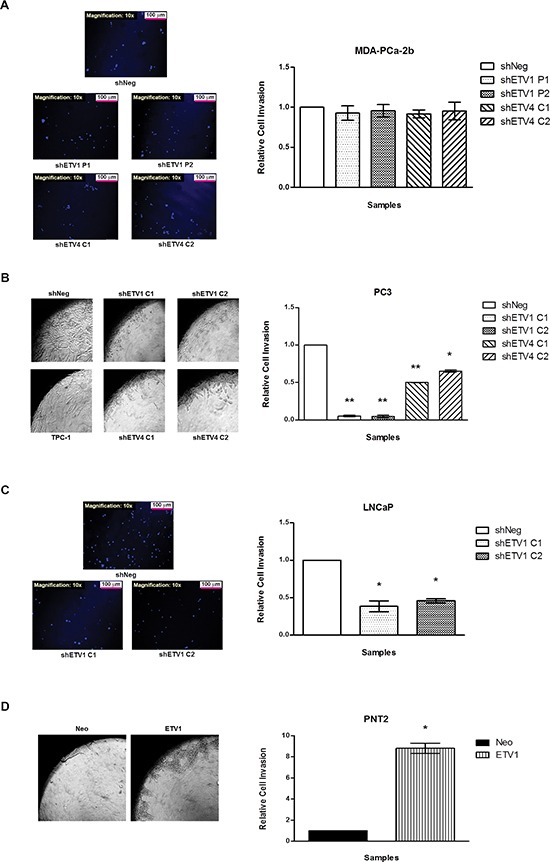
*In vitro* evaluation of the impact of ETV1 or ETV4 silencing in cell invasion **(A–D)** Qualitative visualization (*left*) and quantitative analysis (*right*) of the several cell line models with manipulated *ETV1* or *ETV4* expression: MDA-PCa-2b **(A)** PC3 **(B)**, LNCaP **(C)** and PNT2 **(D)** cells. Results are shown for each silenced cell population relative to the shNeg cells from three independent experiments. Statistically significant *p* values are showed by an asterisk (*p < 0.05; **p < 0.01).

We conducted *in vitro* invasion assays to evaluate the role of *ETV1* or *ETV4* in the ability of MDA-PCa-2b, PC3, LNCaP and PNT2 cells to invade through a basement membrane matrix. MDA-PCa-2b-shNeg cells revealed low invasive capacity, and knockdown of either *ETV1* or *ETV4* did not change that characteristic (Figure 4A). On the other hand, a significant decrease in cell invasion was observed in both shETV1 and shETV4 cells derived from the PC3 cell line, with a much stronger decrease seen in shETV1 cells (80–90% decrease in shETV1 cells *versus* 40–50% decrease in shETV4 cells, Figure 4B). In LNCaP cells, silencing of *ETV1* expression led to a significant 50–60% decrease in invasion when compared to the shNeg control (Figure 4C), whereas in PNT2 cells, *de novo* overexpression of *ETV1* induced an inverse effect, leading to a ten-fold increase in cell invasion when compared to the PNT2-Neo control cells (Figure 4D).

### Whole transcriptome expression profile of *in vitro* cell models reveals candidate PEA3 targets in rearrangement-positive tumors

**Figure 5:**
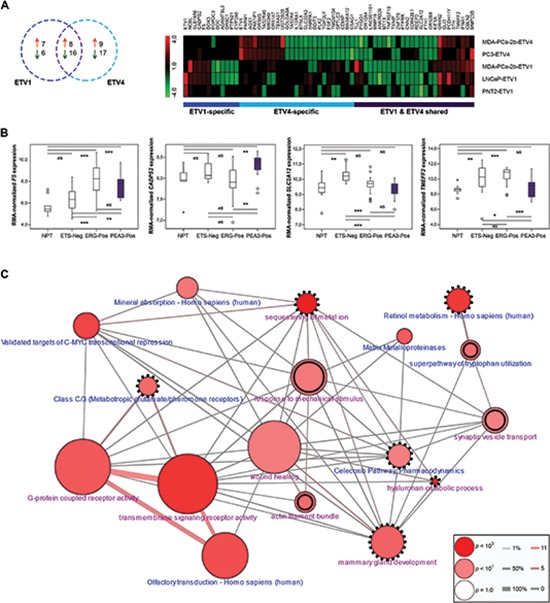
Dissection of ETV1 and/or ETV4 regulated genes **(A)** Venn-diagram (*left*) and hierarchical clustering (*right*) of the 61 ETV1 and/or ETV4 associated genes *in vitro*, showing specific and shared candidate targets (Supplementary Table 1). *ETV1* and *ETV4* are also included. **(B)** Box-plot distribution of the expression of the four ETV1 and/or ETV4 associated genes *in vitro* that discriminate PEA3 positive prostate carcinomas from other tumors or NPT samples. *NS* - not significant, (*p < 0.05; **p < 0.01; ***p < 0.001). **(C)** Gene set enrichment network of Gene Ontology Categories (in *pink*) and Molecular Pathways (in *blue*) for the panel of 61 ETV1 and/or ETV4 associated genes *in vitro* (Supplementary Table 2). Specific enrichment of ETV1 target genes is shown in double-lined circles, specific enrichment of ETV4 target genes in dotted circles and enrichment of both ETV1 and ETV4 targets in simple-lined circles. Connector line thickness represents the percentage of genes shared between categories and line color-code represents the number of shared genes in the input list. Circle size reflects the number of genes in the category and color-grading represents *p* value of enrichment in the input list.

We generated an integrated transcriptomic analysis to gain mechanistic insights into the effect of *ETV1* and *ETV4* in prostate carcinogenesis, and to identify putative target genes. We identified 61 genes that were differentially expressed upon *ETV1* or *ETV4* knockdown (fold-change higher than 1.5 or lower than –1.5) (Figure 5A). A set of genes was found associated with *ETV1* overexpression, with seven genes showing increased expression and six genes showing decreased expression (Supplementary Table 1). On the other hand, the expression of nine and 17 genes was found to be positively and negatively associated with *ETV4* overexpression, respectively (Supplementary Table 1). These sets included two genes (*TLL1* and *PYGO1*) that were present in both *ETV1*- and *ETV4*-specific putative target genes, but their expression was inversely regulated by these ETS members, being up-regulated by *ETV1* and down-regulated by *ETV4*. A set of genes was associated with both ETS when considering that the same fold-change was present in three of the four cell line models, which included eight up-regulated genes and sixteen down-regulated genes (Supplementary Table 1). To evaluate whether any of these genes was differentially expressed in PCa harboring PEA3 rearrangements, we used our data from a series of 50 PCa and nine NPT samples [23]. We found four genes that show association with PCa harboring PEA3 rearrangements (Figure 5B). *F5* and *SLC2A12* can differentiate PEA3-positive from ETS-negative tumors (*p* < 0.01 and *p* < 0.001, respectively) while *CADPS2* and *TMEFF2* can differentiate PEA3-positive from ERG-positive tumors (*p* < 0.01 and *p* < 0.001, respectively).

### Gene set enrichment analysis shows enrichment of PEA3 specific and shared GO categories of genes

The functional gene ontology (GO) evaluation considered around 95% and 90% of the genes in the ETV1 and ETV4 gene sets, respectively, to be involved in at least one GO category. On the other hand, 63% of the genes (both of ETV1 and ETV4 genes sets) are described to be present in at least one pathway. The major enrichment (both in GO annotation and pathway analysis, Supplementary Table 2) involves genes that codify membrane receptors associated with olfactory compounds, with three genes being deregulated by both ETS transcriptions factors (*OR10A4*, *OR52E8*, *OR4N2*) and one (*OR6C1*) and two (*OR5M3* and *OR51A7*) genes of the same family being specifically deregulated by ETV1 and ETV4, respectively (Figure 5C). Other PEA3-shared enriched pathways include the deregulation of matrix metalloproteinases (*MMP23B* and *MMP16*) and of validated targets of C-MYC transcriptional repression (*FTH1* and *TMEFF2*). On the other hand, ETS-related pathway analysis revealed PEA3-specific deregulation of metabolic pathways, namely the deregulation of the tryptophan degradation pathway by ETV1 and the deregulation of the retinol metabolism pathway by ETV4 (Supplementary Table 2). Other PEA3-specific gene ontology categories include the ETV4-specific regulation of the expression of genes associated with sequestering of metal ion (*DDIT3* and *FGF2*) and of two additional members (as the olfactory receptors mentioned before) of the family of G-protein coupled receptors (GPCR) associated with taste recognition (*TAS2R4* and *TAS2R46*) and classified as metabotropic glutamate/pheromone receptors.

### Interaction network analysis reveals ETS specific key players of identical signaling networks

**Figure 6:**
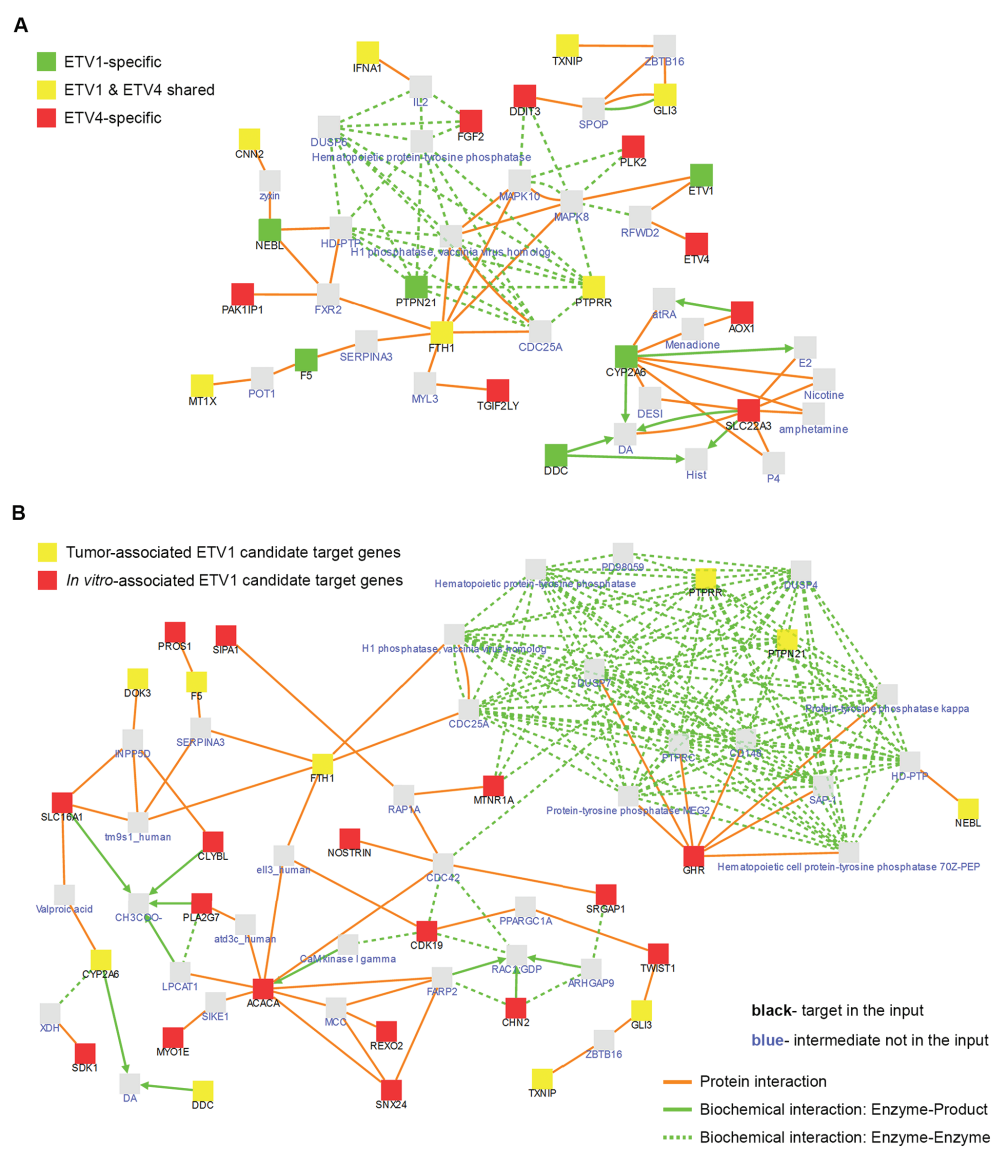
Dissection of ETV1 and/or ETV4 interaction networks **(A)** Interaction networks of ETV1 and/or ETV4 associated genes *in vitro* and known intermediate partners (Supplementary Table 1). **(B)** Interaction networks of *in vitro* and *in vivo* ETV1 associated genes and known intermediate partners (Supplementary Table 3). Proteins from the input list are named in black with color-coded squares and known intermediates not in the input list are named in blue with grey squares. Connector lines are color-coded for the type of interaction: protein interactions in *orange* and biochemical interactions in *green*.

Using the full list of 61 genes associated with ETV1 and ETV4 *in vitro* in the “induced network module” tool of the ConsensusPathDB database, seven genes were not mapped to any protein, one ETV1-specific (*POPDC3*), two ETV4-specific (*MROH9*, *PARM1*) and four shared by both ETS (*ANKRD29*, *LINC00161*, *PCDH11Y*, *STH*). When the remaining 54 PEA3-associated target genes were analyzed together with ETV1 and ETV4, five ETV1-specific (CYP2A6, DDC, F5, NEBL and PTPN21), seven ETV4-specific (AOX1, DDIT3, FGF2, PAK1IP1, PLK2, SLC22A3 and TGIF2LY) and seven PEA3-shared (CNN2, FTH1, GLI3, IFNA1, MT1X, PTPRR and TXNIP) targets showed known protein or biochemical interactions by shared intermediate partners (Figure 6A). When we crossed the ETV1 gene set here identified (n = 38) with our previously identified list of tumor-associated ETV1 target genes (n = 43) no gene overlap was seen (Supplementary Table 3), but ten (26.3%) of the *in vitro*-associated ETV1 candidate target genes and 17 (39.5%) of the tumor-associated ETV1 candidate target genes showed interaction with shared intermediate partners (Figure 6B).

### Combined evaluation of the phenotypic impact and gene expression profile reveals candidate players of invasion and anchorage-independent growth associated with clinico-pathological characteristics of tumor aggressiveness

**Figure 7:**
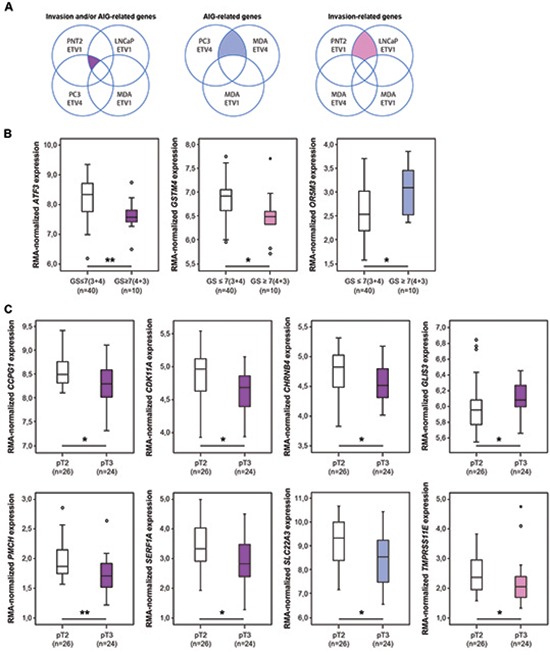
Association between the expression of invasion and/or AIG-related genes *in vitro* and prognostic factors *in vivo* **(A)** Schematic representation of the cell line models used to define invasion and/or AIG-related genes *in vitro*. *Left*, venn-diagram of genes associated with invasion and/or AIG; *center*, venn-diagram of genes associated with AIG; *right*, venn-diagram of genes associated with invasion. **(B)** Box-plot distribution of the expression of the three invasion and/or AIG-related genes associated with higher Gleason score (GS = 7(4 + 3)). **(C)** Box-plot distribution of the expression of the eight invasion and/or AIG-related genes associated with invasive tumors (pT3). The color of the box-plots refers to the venn-diagram from where each gene was obtained. Statistically significant *p* values are showed by an asterisk between box-plots (*p < 0.05; **p < 0.01).

Considering the absence of an overlap between *ETV1* target genes *in vitro* and *in vivo*, we questioned whether we could use the observed impact of *ETV1* and *ETV4* silencing in invasion and AIG to find phenotype-associated genes that could be useful as markers of tumor aggressiveness *in vivo*. Crossing the gene expression profiles of the cell line models showing impact in invasion and/or AIG (Figure 7A) we defined a set of 81 genes with potential to be involved in these oncogenic properties (Supplementary Table 4). A set of 17 genes was considered to be more associated with AIG and a set of 27 genes with invasion. Looking for clinico-pathological associations using the expression profile of our series of 50 prostate carcinomas [23], we found two genes (*ATF3* and *GSTM4*) significantly decreased (*p* = 0.006 and *p* = 0.015, respectively) and one gene *(OR5M3*) significantly increased (*p* = 0.020) in prostate carcinomas with higher Gleason score (GS ≥ 7(4 + 3)) relative to prostate carcinomas with lower Gleason score (GS ≤ 7(3 + 4)) (Figure 7B). On the other hand, seven genes (*CCPG1*, *CDK11A*, *CHRNB4*, *PMCH*, *SERF1A*, *SLC22A3* and *TMPRSS11E*) were found significantly decreased (*p* = 0.046, *p* = 0.012, *p* = 0.016, *p* = 0.006, *p* = 0.044, *p* = 0.021 and *p* = 0.040, respectively) and one gene (*GLIS3*) significantly increased (*p* = 0.042) in pT3 invasive tumors relative to organ confined pT2 tumors (Figure 7C). All these associations were independent of age at diagnosis and PSA at diagnosis. We further explored the clinical utility of this panel of genes in predicting locally invasive tumors combining different expression cut-offs (Table 1). Tumors with decreased expression (bottom 25%) of at least two of these genes (*CCPG1*, *CDK11A*, *CHRNB4*, *PMCH*, *SERF1A*, *SLC22A3* and *TMPRSS11E*) show the strongest correlation with local invasion (*p* = 0.00005). *GLIS3* was excluded from the gene panel for its overlapping distribution in pT2 and pT3 tumors.

**Table 1:** Performance of the panel of seven genes as biomarkers for the identification of tumors with local invasion using different cut-off values

Cut-off (genes in cut-off)	*p* value	PPV	NPV	Sensitivity	Specificity
**Percentil 10 (1 gene)**	0.00017	77%	76%	71%	81%
**Percentil 25 (2 genes)**	**0.00005**	**76%**	**81%**	**79%**	**78%**
**Percentil 50 (4 genes)**	0.00292	67%	75%	75%	67%

## DISCUSSION

Overexpression of oncogenic ETS transcription factors has been associated with chromosomal rearrangements that result in the formation of gene fusions in 50–70% of the prostate cancers [2, 33, 34]. However, information about the biological role of these ETS transcription factors and their downstream target genes remains insufficient. Screening a panel of prostate cancer cell lines for the expression of the five ETS genes described to be involved in genomic rearrangements in PCa (*ERG*, *ETV1*, *ETV4*, *ETV5* and *FLI1*), we found two cell lines – MDA-PCa-2b and PC3 – showing co-overexpression of *ETV1* and *ETV4*. In fact, a similar profile was reported by Hollenhorst *et al*. for PC3 cells [27], but *ETV1* was not considered as overexpressed and the MDA-PCa-2b cell line was not evaluated. Using the expression data of our series of prostate carcinomas subtyped for ETS rearrangements [6], we show that both PC3 and MDA-PCa-2b cells have co-overexpression of *ETV1* and *ETV4*, both ETS in higher levels than those of *ERG* in VCaP cells, the *in vitro* model of the *TMPRSS2-ERG* rearrangement [12]. MDA-PCa-2b and PC3 cells were thus used as suitable models to investigate whether *ETV1* and *ETV4* have similar mechanistic and functional involvement in tumor biology. A recent study selected the DU-145 prostate cancer cell line as a model to address the oncogenic role of *ETV4* in prostate cells [28]. Although this cell line was not included in our initial panel, it could be a valuable model to study the individual role of *ETV4* overexpression in prostate carcinogenesis, which led us to acquire DU-145 from DSMZ and perform qRT-PCR and western blot for both ETS. Our data, however, revealed no *ETV4* (or *ETV1*) overexpression at mRNA or protein levels in DU-145 (not shown), eventually reflecting differences in the DU-145 cell lines studied between the two groups. Looking for the presence of chromosomal rearrangements that might have occurred within the *ETV1* and *ETV4* genes in PC3 and MDA-PCa-2b cells, and contrarily to what was reported by Hollenhorst *et al*. [27], we were able to identify a structural genomic alteration in the PC3 cell line that consists in the presence of the 5′ region of the *ETV4* gene in two similar aberrant chromosomes, in addition to three chromosomes 17 with no rearrangement. Bearing in mind the negative nature of PC3 cells for androgen receptor expression [35], androgens are excluded as possible mediators of *ETV4* overexpression, and the regulatory mechanism remains elusive. As for the MDA-PCa-2b cells, no genomic rearrangement was found to mediate the aberrant overexpression of *ETV4*. Consistent with earlier work [26], an *ETV1* rearrangement was found in the MDA-PCa-2b cell line, validating its authenticity and the cause of the *ETV1* outlier expression. Considering ETS rearrangements as primary events, it is possible that in PC3 cells the structural rearrangement of *ETV4* preceded altered *ETV1* expression, while in MDA-PCa-2b cells the *ETV1* rearrangement might have occurred before *ETV4* overexpression.

In order to gain insight into the phenotypic role of each ETS for prostate carcinogenesis, several *in vitro* experiments were performed. When evaluating the effects of *ETV1* and *ETV4* silencing in AIG by the colony-forming assay, we observed that *ETV4* knockdown strongly impaired the ability of PC3 and MDA-PCa-2b cells to grow in an anchorage-independent way, significantly decreasing the number of colonies formed. These results match those reported by Hollenhorst *et al*. in PC3 cells and are consistent with data reported by Pellecchia *et al*. in DU-145 cells [27, 28], suggesting that high *ETV4* expression levels are necessary for robust AIG. Surprisingly, loss of this property was not observed after depletion of *ETV1* in the same cell line models, contrasting with the phenotype observed after knockdown of *ETV1* in the LNCaP cell line and after *de novo* expression of *ETV1* in the benign PNT2 cells (in line with other reports [25, 36]). Altogether, it is thus reasonable to hypothesize that downregulation of *ETV1*, although not sufficient to revert the cells' ability to form colonies in a co-expression context, appears to be a critical factor when present as a uniquely overexpressed ETS, presumably reflecting an overlapping function with *ETV4*. This hypothesis could also explain the observed impact in apoptosis of *ETV1* silencing in the LNCaP cell line (although not reaching statistical significance), while no impact was observed in the co-expressing cell line models. However, and although we did not find a cell line model uniquely overexpressing *ETV4*, Pellecchia *et al*. (2012) reported no impact in apoptosis after *ETV4* silencing in their DU-145 cells [28], suggesting that *ETV1* involvement in apoptosis may be specific of LNCaP cells, instead of being masked by an *ETV4* overlapping function in MDA-PCa-2b and PC3 cells. Studies in other tumor types also support an involvement of *ETV1* and *ETV4* in AIG [37, 38], but no reference was made regarding the comparative expression of these ETS in the cell models used. Our data, however, point to a stronger involvement of *ETV1* in the acquisition of invasive properties. In fact, although a significant decrease of cell invasion was observed after knockdown of either *ETV1* or *ETV4* in PC3 cells, the global effect of *ETV1* knockdown in cell invasion was much more dramatic than the effect of *ETV4* knockdown in this cell line model. Moreover, a significant gain of cell invasion was obtained by *de novo* overexpression of *ETV1* in the benign PNT2 cells, while a significant decrease was observed after *ETV1* knockdown in the malignant LNCaP cells. These results are consistent with the invasive growth promoting effect described for *ETV1* and *ERG* by other groups [12, 24]. The lack of impact of silencing of either *ETV1* or *ETV4* in the invasive properties of the MDA-PCa-2b cell line may either reflect overlapping functions of these ETS or the existence of other critical factors that promote an invasive phenotype in this cellular context. Consistent with this, Pellecchia *et al*. (2012) reported that, while silencing of *ETV4* in DU-145 cells did not impair cell invasion, *de novo* expression in the nonmalignant RWPE cells lead to a gain of this oncogenic characteristic [28].

Aiming to find molecular players of either ETV1 or ETV4 regulation, we analyzed expression changes between the *in vitro* manipulated cell populations and their respective controls using whole transcriptome exon-level expression arrays. As it has been reported for other ETS transcription factors, and in line with what we have observed concerning the phenotypic impact of *ETV1* and *ETV4*, we found that these members of the PEA3 subfamily of ETS transcription factors are involved in the regulation of both specific and shared molecular partners/pathways. The most surprising data from the gene set enrichment analyses is the deregulation of six members of the family of olfactory receptors, either PEA3 specific or PEA3 shared. Moreover, when searching for phenotype-associated gene expression changes three additional member of this family were found (Supplementary Table 4) and one, *OR5M3*, showed to be associated with higher Gleason score, with potential to be used as a biomarker. The involvement of this type of receptors in cancer development in general has been scarcely explored. In fact, one of the first reports comes from Xu and collaborators with the description of the overexpression of *OR51E2*, first called PSGR (prostate-specific G-protein-coupled receptor), in around 60% of the prostate carcinomas [39], the same gene that we have recently found to be a 5′ fusion partner of *ETV1* [7]. In 2009, Neuhaus and collaborators reported beta-ionone as an activator of OR51E2, suggesting the potential usefulness of specific receptor ligands as therapeutic approaches [40]. Interestingly, the ETV4-specific gene set also revealed deregulation of two taste receptors, members of a largely unexplored subfamily of G protein-coupled receptors (GPCR) that together with olfactory receptors account for over half the GPCR repertoire [41]. As GPCR are cell surface receptors and can be activated by a plethora of stimuli, from photons to peptides, hormones and lipids [41], studies focusing in the search for specific ligands of these olfactory and taste receptors could reveal potential therapeutic approaches for tumors harboring overexpression of these PEA3 members [42]. Other PEA3 deregulated genes include the “deregulation of matrix metalloproteinases” and of “validated targets of C-MYC transcriptional repression”, two GO terms familiar to ETS involvement in prostate carcinogenesis. Despite the *in vitro* association between the expression of both *ETV1* and *ETV4* and the expression of *MMP16* and *MMP23B*, we found no correlation in prostate carcinomas harboring PEA3 rearrangements, suggesting a cell type and microenvironment specific regulation of MMPs [43]. Of the genes included in the GO category of “validated targets of C-MYC transcriptional repression”, *TMEFF2* is worth to be explored. *TMEFF2* codifies an androgen-regulated transmembrane protein, normally restricted to the brain and prostate tissues, initially described as a tumor suppressor gene for its inhibition of cell growth and hypermethylated status in several tumors, including prostate carcinomas [44, 45]. Conversely, other reports showed that a significant proportion of prostate carcinomas exhibit *TMEFF2* overexpression [46, 47] and *in vivo* studies using LNCaP xenografts show that *TMEFF2* inhibition results in tumor growth arrest [46], favoring its role as an oncogene. While this oncogenic prostate-specific role of *TMEFF2* is in line with our observed positive association between *TMEFF2* and *ETV1* and *ETV4* expression, being significantly decreased with silencing of either ETS, our panel of PCa subtyped for ETS rearrangements [23] showed an association of *TMEFF2* overexpression with non-PEA3-positive PCa. This dual activity can result from different *TMEFF2* isoforms, produced from either alternative splicing or cleavage of the extracellular domain, which trigger different signaling cascades [48–51]. Considering the PEA3-rearrangement context and the androgen-regulated nature of the *TMEFF2* gene, it would be interesting to evaluate how the activity of androgens can modulate the expression, subcellular localization and signaling of *TMEFF2* isoforms under PEA3 overexpression. This discrepancy between the observed *in vitro* and *in vivo* expression profiles is also patent in the absence of shared candidate targets between *in vitro* ETV1-associated genes here identified and the *in vivo* ETV1-associated genes previously identified [23]. However, as shown by the interaction network analysis, several of the *in vitro* ETV1 candidate target genes may act in similar pathways as the tumor-associated ETV1 candidate target genes by shared intermediate partners, once again suggesting the importance of the environmental factors and of the cellular context in the activity of key molecular players, such as the ETS transcription factors.

Despite the consistent reports on the involvement of either ETV1 or ETV4 in invasion and AIG *in vitro*, and comparing to what is accepted for other tumor types [42, 52], validation of the contribution of these PEA3 members to an increased aggressiveness of prostate tumors is only emerging. In fact, very few reports clearly suggested that association. In 2008, Attard *et al*. described *ETV1* rearrangements in 23 cases, which were shown to be associated with higher pathological staging, Gleason score and PSA [9]. In 2012, Shaikhibrahim *et al*. reported that *ERG* and *ETV1* are up-regulated in the glands of the peripheral zone comparing with the transitional zone of the prostate, and that PEA3 rearrangements can occur *de novo* in metastatic lesions of rearrangement-negative primary tumors (contrarily to *ERG* rearrangements) [30, 53]. Searching for additional validation, we used data from GSE26242 to look for differentially expressed genes using the interactive web tool GEO2R from the National Center for Bioinformatics (NCBI) [54]. In this cohort, *ETV1* and *ETV4* expression, but not *ERG*, were associated (*p* < 0.05) with higher pathological staging ( ≥ pT3 *versus* ≤ pT2), with *ETV1* also associated with higher Gleason score (GS ≥ 8 *versus* GS ≤ 6) (Supplementary Figure 1). Nevertheless, even though one can prove that a specific gene is regulated by a certain transcription factor, the specificity of that regulation is less plausible, as the same gene can be regulated by other proteins or transcription factors, depending on the cellular context. So, we questioned whether we could use the *in vitro* cellular models with impact in AIG and invasion to find molecular players that may play a role in tumor aggressiveness *in vivo*, independently of the ETS background. By crossing the expression profiles of the cellular models that showed the same phenotypic impact and excluding those that did not, we defined lists of candidate molecular players in AIG and invasion. Interestingly, despite the absence of an overlap between the ETV1-associated *in vitro* and *in vivo* gene sets, two of the genes associated with invasion and/or AIG, namely, *CDK19* and *PROS1*, are present in our *in vivo* gene set, increasing their potential to be involved in the aggressiveness of tumors with ETV1-rearrangement (Supplementary Tables 3 and 4). Although overall the links between the “invasion and/or AIG” genes and prostate cancer or carcinogenesis in general are scarce, the association of decreased *SLC22A3* levels and tumor aggressiveness was previously reported by two groups as part of both an “underexpressed in high Gleason grade signature” and an “underexpressed in progression signature” [55, 56]. Interestingly, *SLC22A3* is also included in our list of ETV4-specific candidate target genes, showing inverse correlation with *ETV4* expression. Since there are no large series of ETV4-positive tumors that can be useful in the evaluation of the prognostic value of ETV4-rearrangements or of the correlation between these and the expression of *SLC22A3*, further studies are necessary to evaluate *SLC22A3* link with *ETV4* and its involvement in AIG. In the study from Tomlins *et al*., decreased *GSTM4* expression was also associated with disease progression as part of the “glutathione metabolism pathway” [55]. Although none of the genes that we found associated with Gleason score and pathological staging in our series was present in the panel of 255 genes evaluated in GSE26242, in the same study Long *et al*. further showed a panel of 304 genes that differentiate tumors with GS = 7(3 + 4) from tumors with GS = 7(4 + 3), where the gene *ATF3*, also identified in our analysis, was described to be significantly decreased in the group of tumors with higher Gleason score [57]. Several other research groups have proposed gene panels that discriminate aggressive from non-aggressive prostate carcinomas [58] and differences between them may be related to several aspects, ranging from characteristics of the sample cohorts to differences in methods and analysis of the data. To our knowledge this is the first study that aimed to translate information from *in vitro* to *in vivo* datasets in the context of PEA3 overexpression. The potential of the panel of seven genes here described as associated with pT3 tumors and their involvement in invasion and/or AIG in the context of PEA3 overexpression warrants further investigation.

In summary, this study provides new information on the oncogenic role of *ETV1* and *ETV4* in prostate carcinogenesis by looking at a co-expression cellular context *in vitro*. We show that these ETS transcription factors have partially overlapping functions, with *ETV1* being more relevant for cell invasion and *ETV4* for anchorage-independent growth. At the molecular level, expression signatures of each ETS reveal the regulation of both specific and shared candidate targets that may resemble cellular mechanisms *in vivo* by interaction with the same intermediate partners. We further identified a panel of genes associated with invasion and anchorage-independent growth mediated by *ETV1* and *ETV4 in vitro* that may be useful as prognostic markers, independently of the ETS rearrangement status. Larger cohorts would be valuable in the validation of these markers and of their link with PEA3 rearrangements, eventually allowing outlining a subset of tumors with worse prognosis.

## METHODS

### Cell culture

PC3 and LNCaP cells were acquired from the German Resource Centre for Biological Material (DSMZ, Braunschweig, Germany). VCaP and PNT2 cells were acquired from The European Collection of Cell Cultures (Sigma-Aldrich, St. Louis, MO). 22Rv1 cells were kindly provided by Dr. David Sidransky at the Johns Hopkins University School of Medicine. MDA-PCa-2b and NCI-H660 cells were kindly provided by Prof. Ragnhild A. Lothe from the Department of Cancer Prevention at the Institute for Cancer Research, Oslo University Hospital, Norway, and TPC-1 cell line was kindly provided by Prof. Paula Soares from the Cancer Biology Group at the Institute of Molecular Pathology and Immunology of the University of Porto. The virus packaging RetroPack™ PT67 cell line was acquired from Clontech Laboratories, Inc. (Saint-Germain-en-Laye, France). Human PC3 cells were grown in F-12 medium, LNCaP, PNT2, 22Rv1, TPC-1 and NCI-H660 cells were grown in RPMI 1640, and VCaP and PT67 cells were grown in DMEM, all from GIBCO, by Life Technologies (Carlsbad, CA). MDA-PCa-2b cells were cultured in BRFF-HPC1 medium (Gentaur, Brussels, Belgium). All media were supplemented with 10% fetal bovine serum (FBS) and 1% penicillin/streptomycin, both from GIBCO, with exception of MDA-PCa-2b cells which were supplemented with 20% FBS, and NCI-H660 cells, supplemented with 5% FBS, 1X Insulin-transferrin-selenium (GIBCO), 2 mM L-glutamine (GIBCO), 10 nM Hydrocortisone (Sigma-Aldrich), and 10 nM beta-estradiol (Sigma-Aldrich). Cells were maintained under appropriate growth conditions. Conventional G-banding karyotyping was performed to confirm cell identity and all prostate cell lines were routinely tested for *Mycoplasma spp*. contamination (PCR Mycoplasma Detection Set, Clontech Laboratories Inc.).

### Taqman low density array (TLDA)

To evaluate the expression of different ETS transcription factors (*ERG*, *ETV1*, *ETV4*, *ETV5* and *FLI1*), all prostate cell lines were analyzed using a TLDA card from Applied Biosystems (by Life Technologies, Foster City, CA) [6]. Briefly, after RNA extraction with TRIzol® Reagent (Invitrogen by Life Technologies), 100ug of RNA were converted to cDNA using the High-Capacity RNA-to-cDNA kit (Applied Biosystems), according to the manufacturer's instructions. The triplicate well format TLDA card included manufacturers' pre-developed probes for *ERG* (Hs01554635_m1), *ETV1* (Hs00951941_m1), *ETV4* (Hs00385910_m1), *ETV5* (Hs00231790_m1), *FLI1* (Hs00956711_m1), *GUSB* (Hs99999908_m1) and *18S* (as pre-included housekeeping control). ETS expression values were obtained by the Comparative Ct method [59], using average *18S* and *GUSB* expression as normalization controls. To validate true ETS outlier expression, ETS expression from each cell line was normalized to the median ETS expression found in our series of prostate carcinomas [6]. Average expression values of 91 PCa with *ERG* rearrangement, 12 with *ETV1* rearrangement, three with *ETV4* rearrangement, one with *ETV5* rearrangement, one with *FLI1* rearrangement and 78 without known ETS rearrangements were used for comparison.

### Fluorescence in situ hybridization (FISH)

To look for genomic rearrangements involving the *ETV1* and *ETV4* transcription factors, we performed FISH in PC3, MDA-PCa-2b and LNCaP cells. Bacterial artificial chromosomes (BACs) were selected using the (UCSC) Human Genome Browser and were obtained from the BACPAC Resource Center (Oakland, CA). For the detection of the *ETV1* gene, the BAC clones used were RP11-941F13 and RP11-790J2 (at 5′ and 3′, respectively), whereas the BACs RP11-259G18 and RP11-831F13 were used to target the *ETV4* gene (at 5′ and 3′, respectively). BAC DNA was isolated, amplified and labeled as previously described [60]. The integrity and correct localization of all probes was verified on normal human metaphases.

### Generation of cell line models

Stable silencing of *ETV1* in LNCaP cells was previously described [23]. The same approach was used for stable silencing of *ETV1* and *ETV4* in MDA-PCa-2b and PC3 cells. Briefly, two shRNA sequences against *ETV1* or *ETV4* were selected and designed using the RNAi Target Sequence Selector and the shRNA Sequence Designer, respectively (Supplementary Table 5). Both shETV1 and shETV4 sequences were cloned into the pSIREN-Retro-Q vector (Clontech Laboratories Inc.). A negative control vector carrying a nontargeting sequence (pSIREN-shNeg) was also generated (Protocol No. PT3132-1, Version No. PR631543). After transfection into the PT67 packaging cell line, viral medium was harvested and used to infect the cells of interest. Stably silenced cell populations were obtained by puromycin selective pressure (1μg/mL, Clontech Laboratories Inc.). The cell populations showing lower *ETV1* or *ETV4* expression levels (evaluated by qRT-PCR, *not shown*) were selected (shETV1-553 cells from MDA-PCa-2b, shETV1-1037 cells from LNCaP and PC3, and shETV4-664 cells from both MDA-PCa-2b and PC3) and clonal populations were isolated using the serial dilutions technique. At least six clonal populations were isolated for each condition and two independent clones showing low levels of the silenced ETS (evaluated by qRT-PCR, *not shown*) were selected for further studies. For each cell line, a control population carrying a non-targeting sequence (shNeg) and two independent low *ETV1* or *ETV4* expressing clones (shETV1-C1 and shETV1-C2, or shETV4-C1 and shETV4-C2, respectively) were used. For the MDA-PCa-2b shETV1 cells, we were not able to establish clonal populations, thus two independent polyclonal populations were selected (shETV1-P1 and shETV1-P2). *De novo* overexpression of *ETV1* in PNT2 cells was previously described [23]. Briefly, the full-length coding sequence of ETV1 (ENST00000242066) was amplified from LNCaP cells and cloned into the pMSCVneo vector (Clontech Laboratories Inc.) using the In-Fusion Cloning System (Clontech Laboratories Inc.). PT67 cells were transfected with pMSCV constructs and with the empty vector pMSCVneo and harvested viral media were used to infect PNT2 cells. Transfected PNT2 cells were expanded under G418 selective pressure (300 μg/mL, GIBCO). A control population (PNT2-Neo) and one cell population showing overexpression of full-length ETV1 (PNT2-ETV1) were used.

### Quantitative reverse transcription PCR (qRT-PCR)

To evaluate the expression levels of *ETV1* and *ETV4* in the established cell line models with silencing of these PEA3 members, total RNA was extracted from all cell populations using the Illustra TriplePrep kit (GE Healthcare, Cleveland, USA) and cDNA was synthesized using the H-minus RevertAid cDNA synthesis kit (Fermentas, St. Leon-Rot, Germany) with oligo-dT primers, according to the manufacturers' protocol. Primers and probes were either acquired as pre-developed TaqMan Gene Expression Assays from Applied Biosystems or designed using the software Primer Express and acquired from Metabion (Martinsried, Germany) (Supplementary Table 6). The beta-glucuronidase (*GUSB*) housekeeping gene was used as an endogenous control for normalization of the expression levels. Relative expression levels of each ETS were obtained by calibrating *GUSB* normalized ETS expression values from each population for the expression levels of the corresponding control population (shNeg).

### Western-blotting

Protein fractions were obtained from sub-confluent cells after RNA extraction with the Illustra TriplePrep kit (mentioned above). Concentration was measured by the Pierce BCA protein assay (Thermo Fisher Scientific, Rockford, USA), according to the manufacturer's instructions. For the immunoblotting, 30 μg of protein extracts were run on a 10% SDS-PAGE gel and blotted onto a Protran nitrocellulose membrane (GE Healthcare). Protein detection was accomplished by overnight incubation at 4°C, using the following primary antibodies: mouse anti-ETV4 (1:500, clone 3G9-1B9, H00002118-M01, Abnova, Atlanta, USA); mouse anti-ETV1 (1:500, clone 4C12, SAB1403794, Sigma-Aldrich) and mouse anti-β-actin (1:8000, clone AC-15, A1978, Sigma-Aldrich) monoclonal antibodies, respectively. An anti-mouse horseradish peroxidase-conjugated secondary antibody was used (1:2500, Santa Cruz Biotechnology, Heidelberg, Germany) and signals were detected using the Immun-Star WesternC Chemiluminescent kit (Bio-Rad Laboratories, Inc., Munich, Germany).

### Proliferation assay

Assessment of cell viability was performed using the MTT assay (Sigma-Aldrich). Cells were seeded onto 96-well plates (Sarstedt, Nümbrecht, Germany) at specific cell densities (Supplementary Table 7) and incubated at normal growth conditions for 24, 48, 72 and 96 hrs. Twelve to 24 hrs after cell seeding (depending on the cell line), a time zero measure was performed, corresponding to the cell viability measure of post-adherence, pre-proliferating, seeded cells. At each time point (including time zero), 100 μL of 0.5 mg/mL MTT solution were added and cells were incubated for 2 hrs (PC3 and LNCaP) or 30 min (MDA-PCa-2b). Formazan crystals were solubilized with 50 μL of dimethyl sulfoxide (DMSO, Merck). The optical density was measured using a microplate reader (Fluostar Omega, BMG Labtech, Ortenberg, Germany) at a wavelength of 540 nm with background correction at 630 nm. For each time-point, an average value of measures from nine replicate wells was obtained. Cell population growth was estimated by correcting and normalizing the average absorbance values obtained in each time-point (Tn) to the average absorbance values of the time zero (T0) by the following formula: (Tn-T0)/T0. Relative growth was obtained by normalizing values of each silenced cell population to its respective control. Three independent assays were performed.

### Apoptosis assay

Apoptosis was analyzed using the APOPercentage apoptosis assay kit (Biocolor, Carrickfergus, UK) according to the manufacturer's instructions. Cells were seeded onto 96-well plates (Sarstedt) at specific cell densities (Supplementary Table 7) and incubated at normal growth conditions for 96 hrs. The absorbance was determined using a microplate reader (Fluostar Omega) at 550 nm with background correction at 620 nm. An average value of measures from nine replicate wells was obtained for each cell population. Relative apoptosis was obtained by normalizing values of each silenced cell population to its respective control. Three independent assays were performed.

### Anchorage-independent growth (AIG)

AIG was measured using the soft agar colony formation assay. A 0.6% bottom layer of low melting point agarose (Lonza by VWR, Radnor, EUA) in normal growth medium was prepared in six-well culture plates (Sarstedt). On top, a layer of 0.2% agarose containing cells (Supplementary Table 7) was placed and covered with culture medium. Cells were incubated at normal growth conditions for two to three weeks. Photographs of representative fields were taken and colonies containing more than seven cells were considered for counting. Relative aggregation was obtained by normalizing values of each silenced cell population to its respective control. Three independent assays were performed.

### Invasion assay

Cell invasion was evaluated using the Oris^TM^ Cell Invasion & Detection Assay kit (Platypus Technologies, Madison, USA) and the BD BioCoat^TM^ Matrigel^TM^ Invasion Chamber (BD Biosciences), according to the manufacturer's recommendations. The first was used with PC3 and PNT2 derived cells, whereas the second was used with LNCaP derived cells. Both assays were used with MDA-PCa-2b derived cells and with the TPC-1 cell line, as a positive control. For the Oris^TM^ Cell Invasion assay, cells were seeded at specific cell densities (Supplementary Table 7) and incubated at normal growth conditions for eight days. At this time-point, cells were stained with Calcein AM (AMS Biotechnology, Abingdon, UK) at a final concentration of 0.5 μg/mL. A detection mask was attached to the plate bottom to restrict visualization of the detection area and measurements were made in a microplate reader (Fluostar Omega). For the Boyden chambers, cell suspensions in serum-free medium (Supplementary Table 7) were loaded in the upper chambers and complete medium was added to the lower chamber. After 48 hrs incubation at normal growth conditions, the non-invading cells attached to the upper surface were removed with cotton swabs, and invading cells on the lower side of the membrane were stained with DAPI and visualized in a fluorescence microscope (Olympus Corporation, Tokyo, Japan). Photographs of representative fields were taken using a 10 x objective lens and the cellSens Dimension software (Olympus Corporation) was used for counting. Relative cell invasion was obtained by normalizing values of each silenced cell population to its respective control. Three independent assays were performed.

### Gene expression profile

The GeneChip Human Exon 1.0 ST arrays were used to assess whole-transcriptome expression levels of the different cell line models. The expression profile of each cell population was obtained after background correction and normalization in the Affymetrix Expression Console v1.1 software, using the Robust Multichip Average (RMA) algorithm. Considering the lower expression of *ETV1* in PC3 cells comparing with those of MDA-PCa-2b and LNCaP cells, the shETV1-PC3 cells were not evaluated. Two independent populations of the PNT2-ETV1 cells were evaluated together with the PNT2-Neo control. For each gene and each cell line, two fold-change values, from two manipulated cell populations *versus* control, were obtained. Whenever a gene showed the same differential expression profile (increased or decreased with ETS expression) in both manipulated cell populations, the average fold-change was calculated and the gene was considered for subsequent filtering.

### Identification of specific and shared ETV1 and ETV4 target genes *in vitro*

To identify ETV1 and/or ETV4 candidate target genes, the expression profiles of the cell populations sharing the overexpressed ETS were used. Considering the non-tumorigenic nature of the PNT2 cell line, the expression profile of the PNT2-ETV1 cells was not considered for target filtering. To look for ETS-specific candidate target genes we selected those that showed to be up or down-regulated no less than 1.5-fold in the two cell line models where the expression of the same ETS was silenced (LNCaP and MDA-PCa-2b cells for ETV1, and PC3 and MDA-PCa-2b cells for ETV4). As for the target genes shared by ETV1 and ETV4, we selected those that showed to be up or down-regulated at least 1.5-fold in three of the four silenced cell lines (LNCaP-shETV1, MDA-PCa-2b-shETV1, PC3-shETV4 and MDA-PCa-2b-shETV4).

### Gene set enrichment and interaction network analyses

To explore the functional classification of the candidate target genes obtained by differential expression analysis (as explained above) we used the ConsensusPathDB, which integrates protein interaction information from 32 public interaction databases (Release 29, 27.06.2014) [61–63]. To evidence specific and shared features of ETV1 and ETV4 candidate target genes, analyses of enrichment of “gene ontology categories” and “gene involvement in known signalling pathways” were performed with the full list of PEA3 candidate targets (n = 61). A cut-off *p* value of 0.02 was used in both analyses. To search for pathway-based sets the information from all ConsensusPathDB integrated databases was considered. To explore possible protein-protein interaction networks associated with the gene sets identified *in vitro*, the “induced network module” tool of the ConsensusPathDB database was used. High and medium-confidence, binary and complex, protein-protein interactions and biochemical reactions were selected for filtering. Considering our previous work focusing on the identification of candidate target genes of ERG and ETV1 using a panel of PCa subtyped for ETS rearrangements [23], we questioned whether *in vitro* and *in vivo* ETV1 candidate targets would overlap or be involved in the same signaling pathways. We thus performed the interaction network analysis using the full list of ETV1 candidate target genes *in vitro* and *in vivo* (Supplementary Table 3).

### Identification of invasion and/or AIG associated genes *in vitro*

To find gene sets associated with the observed impact of ETV1 and ETV4 overexpression in invasion and AIG that may be useful as markers of tumor aggressiveness *in vivo* we crossed the gene expression profiles of the cell line models showing impact in these phenotypic characteristics. To identify genes involved in invasion and/or AIG, we looked for genes that showed the same differential expression profile in the three cell line models showing equal impact in both phenotypic characteristics – shETV4-PC3, shETV1-LNCaP and PNT2-ETV1 cell lines (fold-change higher than 1.2) – and excluded those differentially expressed in the cell line model showing no impact in both phenotypes (shETV1-MDA-PCa-2b cells, fold-change higher than 1.2). Since we observed more involvement of ETV4 in AIG, we looked for genes with higher potential to be involved in this phenotypic characteristic by selecting those that were differentially expressed (fold-change higher than 1.5) in the shETV4 populations of both the MDA-PCa-2b and PC3 cells and excluded those that showed the same differential expression (fold-change higher than 1.2) in the shETV1 populations of the MDA-PCa-2b cells. Similarly, since we observed more involvement of ETV1 in invasion, we looked for genes with higher potential to be involved in invasion by selecting those that were differentially expressed (fold-change higher than 1.5) in both shETV1-LNCaP and PNT2-ETV1 cell populations, and excluded those that showed the same expression profile (fold-change higher than 1.2) in both the shETV4 and shETV1 populations of the MDA-PCa-2b cells.

### *In vivo* validation and clinico-pathological associations

To evaluate whether the observed *in vitro* associations between ETV1 and ETV4 overexpression and the candidate target genes would be useful in the identification of PCa harboring rearrangements of these ETS members, and to evaluate the usefulness of the sets of genes associated with invasion and AIG in the identification of aggressive prostate carcinomas, we used our exon-array expression data from a series of nine noncancerous prostate tissues (NPT) and 50 prostate carcinomas (PCa) subtyped for ETS rearrangements [23], which include 14 PCa with PEA3 rearrangements (12 of which with ETV1 rearrangement), 22 PCa with ERG rearrangement and 14 PCa without known ETS rearrangements. To search for clinico-pathological associations, information on age at diagnosis, PSA at diagnosis, pathological staging and Gleason score was used [6].

### Statistical analysis

All results from *in vitro* assays are expressed as a ratio of the data obtained with each clone and the scramble control, from three independent experiments, each including triplicate wells per condition. The Students *t*-test was used to assess the significance of the differences. To compare expression levels between ETS groups and to find clinico-pathological associations, the Mann-Whitney test was used. To evaluate the performance of the panel of seven genes as biomarkers for the identification of tumors with local invasion using different cut-off values, the Pearson Chi-square test was used. In every statistical test, a *p* value smaller than 0.05 was considered statistically significant.

## SUPPLEMENTARY FIGURE AND TABLES







## ABBREVIATIONS

AIG: anchorage-independent growth
DMSO: dimethyl sulfoxide
FBS: fetal bovine serum
FISH: fluorescence in situ hybridization
MTT: 3-(4,5-dimethylthiazol-2-yl)-2,5-diphenyltetrazolium bromide
NPT: noncancerous prostate tissues
PCa: prostate carcinoma
qRT-PCR: quantitative reverse transcription polymerase chain reaction
shRNA: short hairpin RNA
TLDA: TaqMan low density array
