# Supplementary material for: Specific and redundant activities of ETV1 and ETV4 in prostate cancer aggressiveness revealed by co-overexpression cellular contexts
Source: Oncotarget. 2015 Feb 14;6(7):5217–36. doi: 10.18632/oncotarget.2847 (PMC4467144; doi:10.18632/oncotarget.2847)
Supplement: Supplementary file 1 [file oncotarget-06-5217-s001.pdf]

SUPPLEMENTARY FIGURE AND TABLES

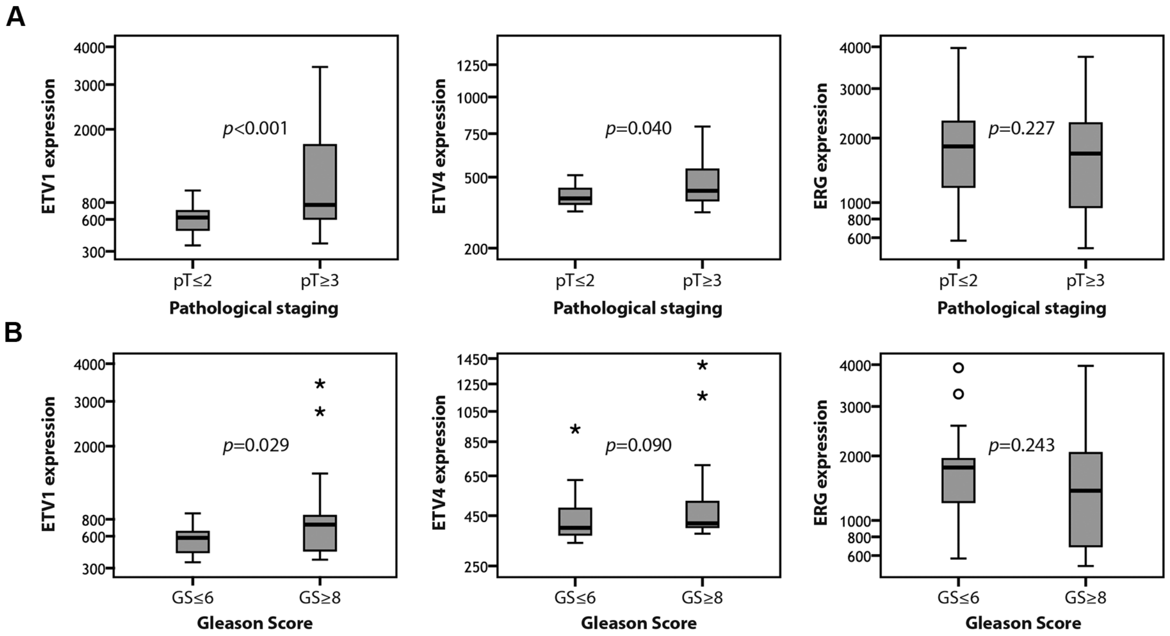

Supplementary Figure 1: Clinico-pathological associations of the expression profile of *ETV1*, *ETV4* and *ERG* obtained from the GEO DataSet GSE26242 (A and B). Box-plot distribution of the expression of *ETV1* (*left*), *ETV4* (*center*) and *ERG* (*right*) according to the Gleason Score **(A)** and pathological staging **(B)**. The *p*-value obtained with the GEO2R analysis is shown for each two group comparison.

Supplementary Table 1: Differential expression values of the 61 genes found associated with *ETV1* and/or *ETV4 in vitro*.

	Probe Set ID	Gene Symbol	MDA-ETV1	LNCaP-ETV1	MDA-ETV4	PC3-ETV4	PNT2-ETV1	Protein Name
ETV1-specific candidate targets	3605395	ADAMTSL3	–1.584	–1.714	1.000	–1.164	–1.310	ADAMTS-like 3
	3070309	CADPS2	1.784	1.642	1.000	1.446	1.000	Ca++-dependent secretion activator 2
	3862873	CYP2A6	–1.803	–2.531	1.000	1.000	1.000	cytochrome P450, family 2, subfamily A, polypeptide 6
	3050388	DDC	–2.873	–1.713	1.000	1.101	1.000	dopa decarboxylase (aromatic L-amino acid decarboxylase)
	2888879	DOK3	1.822	1.514	–1.069	1.000	1.311	docking protein 3
	2443370	F5	1.552	1.554	1.000	1.000	1.000	coagulation factor V (proaccelerin, labile factor)
	3280573	NEBL	3.633	1.792	1.000	1.000	1.306	nebulette
	3416830	OR6C1	–1.557	–2.091	–1.156	1.133	1.000	olfactory receptor, family 6, subfamily C, member 1
	2967276	POPDC3	–1.734	–1.654	–1.236	1.000	–1.162	popeye domain containing 3
	3575302	PTPN21	–1.685	–2.478	1.000	–1.111	–1.099	protein tyrosine phosphatase, non-receptor type 21
	3768412	SLC16A6	1.680	1.789	–1.037	–1.093	1.000	solute carrier family 16, member 6 (monocarboxylic acid transporter 7)
	2522247	AOX1	1.121	–1.111	1.960	1.955	–1.166	aldehyde oxidase 1
	4047607	BTNL8	1.000	1.000	–1.913	–1.571	1.466	butyrophilin-like 8
	3604147	CEMIP	1.056	1.039	–1.542	–2.035	–1.045	cell migration inducing protein, hyaluronan binding
	3995331	CSAG1	1.000	–1.335	–1.870	–3.629	1.092	chondrosarcoma associated gene 1

(*Continued*)

ETV4-specific candidate targets	3458587	DDIT3	–1.299	1.000	–2.098	–1.614	–1.049	DNA-damage-inducible transcript 3
	2742109	FGF2	1.114	1.227	–1.658	–1.773	1.377	fibroblast growth factor 2 (basic)
	3617458	GOLGA8A	1.189	1.000	–1.764	–1.549	1.114	golgi A8 family, member A
	3988538	IL13RA1	–1.083	–1.185	–1.586	–1.558	–1.434	interleukin 13 receptor, alpha 1
	4026206	MAGEA12	–1.230	1.000	–1.694	–2.085	–1.156	melanoma antigen family A, 12
	2366884	MROH9	1.289	–1.346	1.517	1.695	1.000	maestro heat-like repeat family member 9
	3318248	OR51A7	1.371	–1.536	1.564	1.677	1.000	olfactory receptor, family 51, subfamily A, member 7
	3373420	OR5M3	1.000	1.115	1.554	1.827	1.000	olfactory receptor, family 5, subfamily M, member 3
	2894663	PAK1IP1	1.261	–1.034	2.624	1.843	1.440	PAK1 interacting protein 1
	2731636	PARM1	1.000	1.293	1.914	2.078	1.000	DKFZP564O0823 proteina
	2858023	PLK2	1.479	1.098	–1.560	–1.635	–1.569	polo-like kinase 2 (Drosophila)
	2934521	SLC22A3	1.000	1.254	–2.313	–1.578	1.006	solute carrier family 22 (extraneuronal monoamine transporter), member 3
	3642162	SNRPA1	–1.272	1.692	–1.638	–1.587	1.221	small nuclear ribonucleoprotein polypeptide A'
	3876990	SPTLC3	1.674	1.000	–1.577	–2.009	–1.511	serine palmitoyltransferase, long chain base subunit 3
	3027956	TAS2R4	1.095	1.155	–1.789	–1.555	1.000	taste receptor, type 2, member 4
	3444525	TAS2R46	1.000	1.353	1.585	1.706	–1.599	taste receptor, type 2, member 46

(*Continued*)

	3027204	TBXAS1	–1.162	–1.045	1.569	1.666	1.106	thromboxane A synthase 1 (platelet)
	4028649	TGIF2LY	1.000	–1.373	–2.244	–1.703	1.069	TGFB-induced factor homeobox 2-like, Y-linked
	2790062	TMEM154	1.409	1.265	–2.233	–1.829	–1.678	transmembrane protein 154
	2730021	UGT2B28	1.186	–1.133	3.862	1.661	1.185	UDP glucuronosyl transferase 2 family, polypeptide B28
ETV1- and ETV4-shared candidate targets	3801492	ANKRD29	–1.805	–3.860	–1.659	1.000	–1.157	ankyrin repeat domain 29
	3812864	CBLN2	2.451	2.033	1.829	1.000	–1.057	cerebellin 2 precursor
	3815399	CNN2	–1.553	–1.501	–1.651	–1.114	1.154	calponin 2
	3375648	FTH1	–1.444	1.222	–2.005	–1.695	–1.430	ferritin, heavy polypeptide 1
	3047660	GLI3	1.686	1.686	2.104	–1.090	1.035	GLI family zinc finger 3
	3164825	IFNA1	2.036	1.000	1.949	2.252	–1.692	interferon, alpha 1
	3418298	KIF5A	1.626	–1.801	–2.053	–1.590	–1.635	kinesin family member 5A
	3917073	LINC00161	–1.959	–1.523	1.000	–2.121	–1.022	long intergenic non-protein coding RNA 161
	3143643	MMP16	–1.860	–1.798	–1.552	1.000	1.000	matrix metallopeptidase 16 (membrane-inserted)
	4042278	MMP23B	2.727	1.528	1.000	4.925	2.160	matrix metallopeptidase 23B
	3662247	MT1X	–1.692	–1.590	–1.411	1.000	1.000	metallothionein 1X
	3318976	OR10A4	–2.120	1.000	–1.739	–1.572	–1.234	olfactory receptor, family 10, subfamily A, member 4
	3527290	OR4N2	1.518	3.270	3.324	2.168	2.009	olfactory receptor, family 4, subfamily N, member 2

(*Continued*)

	3360687	OR52E8	1.000	–1.444	–1.612	–1.866	1.000	olfactory receptor, family 52, subfamily E, member 8
	4028716	PCDH11Y	1.766	3.352	1.772	1.000	1.000	protocadherin 11 Y-linked
	3461883	PTPRR	–1.554	–1.856	–1.526	1.000	–1.577	protein tyrosine phosphatase, receptor type, R
	3625440	PYGO1	1.756	1.605	–4.872	–1.481	–1.129	pygopus homolog 1 (Drosophila)
	3249043	REEP3	–1.537	–1.632	–1.846	1.286	–1.580	receptor accessory protein 3
	3701779	SDR42E1	–1.550	–1.640	–1.520	1.000	–1.413	short chain dehydrogenase/ reductase family 42E, member 1
	2974935	SLC2A12	–1.521	–2.803	–1.339	–1.407	–4.278	solute carrier family 2 (facilitated glucose transporter), member 12
	3723755	STH	1.768	1.663	1.852	–1.031	1.000	saitohin
	2750753	TLL1	8.179	1.503	–1.990	–1.986	1.000	tolloid-like 1
	2592598	TMEFF2	1.900	1.521	1.000	1.632	–1.165	transmembrane protein with EGF-like and two follistatin-like domains 2
	3481410	TNFRSF19	–1.644	–1.595	–3.423	1.169	–1.251	tumor necrosis factor receptor superfamily, member 19
	2356115	TXNIP	–1.574	1.000	–1.540	–1.519	1.209	thioredoxin interacting protein
	3840286	ZNF578	–1.566	–1.644	–1.180	–1.521	1.000	zinc finger protein 578

Note: Positive values stand for positive associations and negative values for negative associations

Supplementary Table 2: Enriched Gene Ontology Categories and Molecular Pathways of the panel of genes associated with *ETV1* and/or *ETV4 in vitro*.

p-value	GO Term/ Pathway Database	GO Categorie/Molecular Pathway	Number of members in GO Categorie/ Molecular Pathway	% of input in GO Categorie/ Molecular Pathway	Members of input in GO Categorie/Molecular Pathway
0.015	PharmGKB	Celecoxib Pathway, Pharmacodynamics	58	3.448	DDIT3; TBXAS1
0.007	Reactome	Class C/3 (Metabotropic glutamate/pheromone receptors)	40	5.000	TAS2R4; TAS2R46
0.002	Wikipathways	Matrix Metalloproteinases	31	6.452	MMP16; MMP23B
0.006	KEGG	Mineral absorption - Homo sapiens (human)	51	3.922	FTH1; MT1X
0.009	KEGG	Olfactory transduction - Homo sapiens (human)	405	1.235	OR4N2; OR5M3; OR52E8; OR10A4; OR51A7
0.018	KEGG	Retinol metabolism - Homo sapiens (human)	64	3.125	AOX1; UGT2B28
0.005	HumanCyc	**Superpathway of tryptophan utilization**	44	4.545	DDC; CYP2A6
0.002	PID	Validated targets of C-MYC transcriptional repression	75	4.000	TMEFF2; FTH1; DDIT3
0.004	GO:0032432	**Actin filament bundle**	50	4.000	NEBL; CNN2
0.006	GO:0004930	G-protein coupled receptor activity	877	0.798	OR10A4; OR4N2; OR5M3; TAS2R4; OR52E8; TAS2R46; OR51A7
0.001	GO:0030214	Hyaluronan catabolic process	14	14.286	CEMIP; FGF2
0.004	GO:0030879	Mammary gland development	131	2.290	GLI3; ETV4; FGF2
0.005	GO:0009612	**Response to mechanical stimulus**	172	1.744	ETV1; TXNIP; CNN2
0.000	GO:0051238	Sequestering of metal ion	46	6.522	FTH1; DDIT3; FGF2
0.009	GO:0048489	**Synaptic vesicle transport**	72	2.778	DDC; CADPS2
0.010	GO:0042060	Wound healing	672	0.744	GLI3; IFNA1; F5; KIF5A; CNN2
0.001	GO:0004888	Transmembrane signaling receptor activity	1254	0.797	IL13RA1; OR10A4; OR4N2; OR5M3; TAS2R4; TNFRSF19; OR52E8; TAS2R46; PTPRR; OR51A7

Bold- ETV1 enriched Cateories/Pathways; Underlined- ETV4 enriched Categories/Pathways; Simple text- ETV1 and ETV4 shared Categories/Pathways

Supplementary Table 3: *ETV1* candidate target genes *in vitro* and *in vivo*.

Supplementary Table 4: Invasion and/or AIG-associated genes *in vitro*.

Supplementary Table 5: Sequences of the shRNAs designed for *ETV1* and *ETV4*.

Target	Translation start-site (bp)	Sequence 5′-3′ (sense strand)
ETV1	553	GATCCGCTCATACACCGAAACCTGATTCAAGAGATCAGGTTTCGGTGTATGAG TTTTTTACGCGTG
ETV1	1037	GATCCACAAGAGCCAGGAATGTATTTCAAGAGAATACATTCCTGGCTCTTG TTTTTTTACGCGTG
ETV4	664	GATCCAGCAGAGCTTTAAGCAAGATTCAAGAGATCTTGCTTAAAGCTCTGCT TTTTTTACGCGTG
ETV4	940	GATCCGCTGAGAAATTTGAAGGAGATTCAAGAGATCTCCTTCAAATTTCTCAG TTTTTTACGCGTG

Underlined nucleotides- target specific mRNA sequences (sense and antisense)

Supplementary Table 6: Assay ID or sequence of the primers and probes for qRT-PCR

Assay	Assay/Primer name	Assay ID/Primer sequence 5′-3′
qRT-PCR	ETV1	Hs00951941_m1
qRT-PCR	ETV4-PF[a]	GGAATGGAGTTCAAGCTCATTGA
qRT-PCR	ETV4-PR[b]	TAATTCATGGCTGGCCGGTTCT
qRT-PCR	ETV4-PB[c]	CCAGAGCCTGGCGACCTCCTCAG
qRT-PCR	GUSB	4333767F

[a]Primer Forward; [b]Primer Reverse; [c]Probe (5’ FAM and 3’ TAMARA)

Supplementary Table 7: Number of cells used per cell line and per assay

Cell line \ Assay	Proliferation (cells/well)	Apoptosis (cells/well)	AIG[a] (cells/well)	Invasion (cells/well)
shETV1-PC3	2.5×10^3	5×10^3	1×10^5	1×10^4
shETV4-PC3	5×10^3	1×10^4	1×10^5	1×10^4
shETV1-LNCaP	2×10^4	2×10^4	2×10^4	2.5×10^4
shETV1-MDA-PCa-2b	3×10^4	3×10^4	5×10^4	2.5×10^4
shETV4-MDA-PCa-2b	3×10^4	3×10^4	5×10^4	2.5×10^4
PNT2-ETV1	NA	NA	2×10^4	2×10^4
TPC-1	NA	NA	NA	1×10^4

[a]Anchorage-independent growth

Note: For each assay, the shNeg control was analyzed in parallel and the number of cells used was the same as the shETS populations. The number of cells used for each assay was optimized for the growth conditions.
